# Neural Substrate of Initiation of Cross-Modal Working Memory Retrieval

**DOI:** 10.1371/journal.pone.0103991

**Published:** 2014-08-04

**Authors:** Yangyang Zhang, Yang Hu, Shuchen Guan, Xiaolong Hong, Zhaoxin Wang, Xianchun Li

**Affiliations:** 1 School of Psychology and Cognitive Science, East China Normal University, Shanghai, P. R. China; 2 Institute of Cognitive Neuroscience, East China Normal University, Shanghai, P. R. China; University Medical Center Goettingen, Germany

## Abstract

Cross-modal working memory requires integrating stimuli from different modalities and it is associated with co-activation of distributed networks in the brain. However, how brain initiates cross-modal working memory retrieval remains not clear yet. In the present study, we developed a cued matching task, in which the necessity for cross-modal/unimodal memory retrieval and its initiation time were controlled by a task cue appeared in the delay period. Using functional magnetic resonance imaging (fMRI), significantly larger brain activations were observed in the left lateral prefrontal cortex (*l*-LPFC), left superior parietal lobe (*l*-SPL), and thalamus in the cued cross-modal matching trials (CCMT) compared to those in the cued unimodal matching trials (CUMT). However, no significant differences in the brain activations prior to task cue were observed for sensory stimulation in the *l*-LPFC and *l*-SPL areas. Although thalamus displayed differential responses to the sensory stimulation between two conditions, the differential responses were not the same with responses to the task cues. These results revealed that the frontoparietal-thalamus network participated in the initiation of cross-modal working memory retrieval. Secondly, the *l*-SPL and thalamus showed differential activations between maintenance and working memory retrieval, which might be associated with the enhanced demand for cognitive resources.

## Introduction

Working memory is a central cognitive function at the interface of perception and action [1]. It allows humans and animals to use information that is not currently available in the environment. It is necessary for complex cognitive tasks such as language comprehension, learning, decision making and reasoning [2]. Fuster and Alexander (1971) first found that prefrontal neurons displayed persistent discharges during the delay period of a delayed-response task only when monkeys successfully maintained the memoranda [3]. The persistent delay activities are selective depending on the features of the memoranda, such as the spatial location [4], object identity [5] and haptic sensation [6]. Such sustained delay activity has been considered to be the neuronal basis for working memory. Memory cells have been repeatedly observed in prefrontal cortex [7], inferior temporal cortex [8], posterior parietal cortex [9] and subcortical structures [10], [11]. Therefore, working memory is associated with a broad network in the brain.

In many cases, working memory requires integration/interaction of different senses [12]. When monkeys were trained to remember a tone of a certain pitch and then choose the color associated with it after delay period, most of prefrontal neurons activated selectively to tones responded to colors according to the association between tone and color. This finding revealed neuronal responses to a tone in prefrontal cortex were correlated with their subsequent reaction to the associated color, while this correlation faltered in error trials [13]. It suggests that PFC is a member of neural networks related to cross-modal associations. More recently, sensory cortices (such as visual cortex and superior temporal syrus) displayed gradually increased activations as subjects learned both an auditory-visual and visuo-auditory paired-association learning tasks. However, these regions did not significantly change their activations as participants acquired a visuo-visual unimodal association task [14]. The findings indicate some sensory cortices are also involved in cross-modal working memory. A recent PET study also revealed that visual cortex of subjects who had previously been exposed to the audiovisual stimuli showed increased activation after presenting with auditory component of audiovisual events, while visual cortex of naive subjects did not significantly change the responses to auditory components [15]. These findings suggest that cross-modal working memory could be represented by the co-activation of the multiple cortical areas in the brain. In order to elucidate how brain initiates memory retrieval of long-term memory, Naya et al. (1996) trained monkeys to perform a pair-association task (PA task) or conventional delayed matching-to-sample (DMS) task according to a color switch in the middle of the delay period [16]. They found that many neurons in anterior inferotemporal cortex (AIT) showed increased discharges just after color switch in the PA task compared to the DMS task. They proposed AIT was involved in the initiation of memory retrieval of long-term memory. However, the neural basis of initiation of cross-modal working memory retrieval remains unknown.

Many pieces of evidence have revealed that different networks in the brain are involved in maintenance and manipulation components of working memory. D’Esposito et al (1999) required participants to retain a sequence of letters (maintenance trial) and reorder the sequence in alphabetical order (manipulation trial) during the delay period of delayed response task. They found that Dorsolateral prefrontal cortex (DLPFC) and ventrolateral prefrontal cortex (VLPFC) enhanced their activations during delay period, but DLPFC displayed significantly higher delay activity in manipulation trials [17]. Therefore, DLPFC exhibit greater recruitment in transformation of information held in working memory. In another fMRI study, Glahn et al (2002) reported superior frontal sulcal area was involved in maintenance spatial information while DLPFC was involved in manipulation of internal representations [18]. However, manipulations in those studies just happened within one kind of modality of information, such as spatial and visual information. Then, which and how brain regions control retaining and manipulating the internal representations held in working memory between different modalities still keep unknown so far.

In the present study, we developed a cued matching task in which the necessity for cross-modal/unimodal memory retrieval and their initiation time were controlled by a task cue appeared in the delay period. Participants were asked to hold sample stimulus (S1, auditory stimulus) in mind till appearance of task cue, then retrieve the associated sensory information (auditory or visual stimulus) according to the task cue, and finally decide whether the attended modality of simultaneous combination of auditory and visual stimuli matched S1 by pressing a button. The cued cross-modal matching trial (CCMT) and unimodal matching trial (CUMT) were presented in a random order in each block. By using this task, we first examined the neural substrates of initiation of cross-modal working memory retrieval by comparison of responses to task cues in CCMT trials with those in CUMT trials. We anticipated that task cue elicited greater activation in frontoparietal network in CCMT trials than in CUMT trials. We secondly investigated whether the differential networks of maintenance and manipulation processes of cross-modal working memory differed from that of unimodal working memory by comparison of activations between intention period (time gap between sample stimulus and task cue) and memory retrieval period (time gap between task cue and matching stimuli).

## Materials and Methods

### Participants

#### Ethics Statement

Ethical approval was obtained from the East China Normal University Internal Review Board. All participants signed their written consent forms before experiment and got certain amount of financial reward as compensation for their time after experiment.

#### Participants

Twenty healthy college students (8 Women, 22–26 years old) participated in this study. All subjects were in good health with no history of psychiatric or neurological diseases. All of them had normal or corrected-to-normal (with contact lenses) visual acuity and could detect the range of auditory frequencies used in our experiment when presented monaurally.

### Stimuli

#### Auditory stimulus

**Figure 1:**
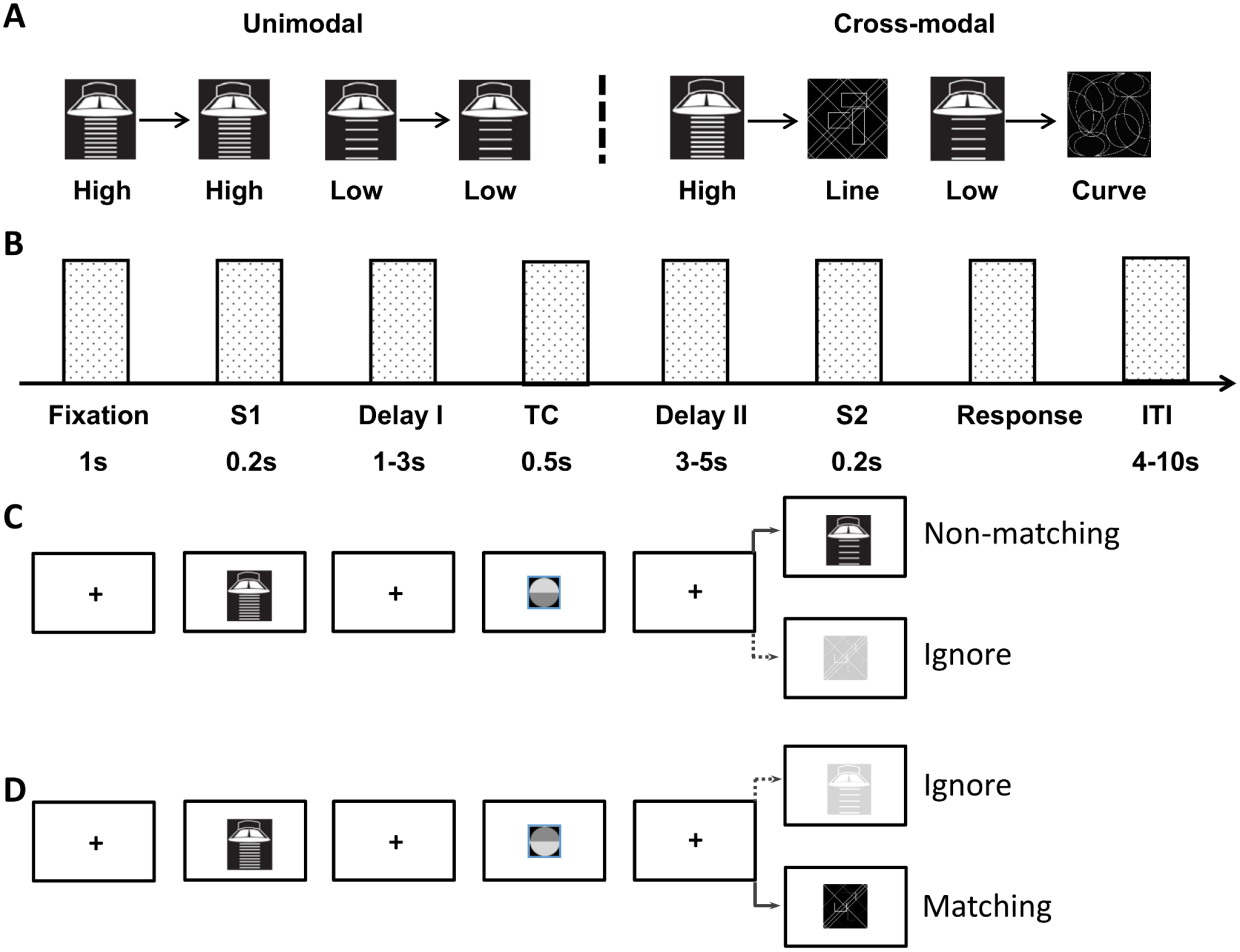
The schematic illustration for the cued matching tasks. ***A***: The matching stimuli in the unimodal working memory task (*left*, high tone - high tone and low tone - low tone) and the crossmodal working memory task (*right*, high tone - line and low tone - curve); ***B***: The events in a cued matching task trial. The solid arrows after task cue indicate the attended stimulus and the dashed arrows indicate the ignored stimulus according to the feature of task cue. ***C***: the cued unimodal matching task (CUMT): S1 (high tone or low tone) with a duration of 200 ms is followed by a TC (a cycle with light-gray in upper half, 500 ms), the auditory (high or low tone) and visual (line or curve) stimuli are simultaneously presented for 200 ms after the Delay II, the participants are asked to attend the auditory stimulus and ignore the visual stimulus, finally they have to report whether the auditory S2 matches the S1 by pressing a button as correctly and quickly as possible. ***D***: the cued crossmodal matching task (CCMT): the sequences of the CCMT are identical to the CUMT except that the task cue is the cycle with dark-gray in upper half. The participants are asked to attend the visual stimulus and ignore the auditory stimulus, and report whether the visual S2 matches auditory S1 by pressing a button.

Two tones with a frequency of either 2 KHz or 0.5 KHz with duration of 200 ms (Fig. 1A) were used as auditory stimuli, and presented dichotically through magnetically compatible headphones.

#### Visual stimulus

Visual stimuli consisting of either line or curve were randomly generated by Matlab7.0 program (Fig. 1A). Each visual stimulus had a size of 2° visual angle with a duration of 200 ms.

#### Task cue

Task cue was a cycle with light-gray in half and dark-gray in the other (Fig. 1). The one with lower dark-gray indicated the ongoing trial was a cued unimodal (auditory-auditory) working memory trial (CUMT) (Fig. 1C). The one with upper dark-gray indicated the ongoing trial was a cued cross-modal (auditory-visual) working memory trial (CCMT) (Fig. 1D). Task cues of CCMT and CUMT were randomly presented in each block. Prior to scanning, all subjects were required to learn the meanings of the task cues.

### Cued matching task

In the cued matching task, subjects performed a CUMT trial or CCMT depending on task cue trial-to-trial (Fig. 1). They were instructed to retrieve the associated sensory information (auditory or visual stimulus) immediately after task cue, and finally decided whether the attended modality of simultaneous combination of auditory and visual stimuli matched S1 according to task cue by pressing a button (i.e., “1” for matching, “2” for non-matching). Subjects completed 4 blocks of the cued matching task, and each block had 16 CCMT trials and 16 CUMT trials. Each block lasted for 6.8 min, and the inter-block interval was approximately 1 min. Thus the total session lasted for approximately 30 min for each subject. The order of the four blocks was counterbalanced across participants.

#### The cued unimodal matching trial (CUMT, Fig 1B&C)

Each trial began with S1 (auditory stimulus, high tone or low tone) with the duration of 200 ms followed by a Delay-I (Duration of 1 s∼3 s with a step of 0.5 s). Participants were asked to memorize the feature of S1 during Delay-I. The task cue (a cycle with dark-gray in lower half) appeared for 500 ms at the end of Delay-I, participants were asked to retrieve the association between S1 and S2 during Delay-II (Duration of 3 s∼5 s with a step of 0.5 s). At the end of Delay-II, combination of auditory (high or low tone) and visual (line or curve) stimuli as S2 were presented for 200 ms, participants were required to attend the auditory stimulus and ignore the visual stimulus, finally to report whether the auditory S2 matched the S1 by pressing a button as correctly and quickly as possible (i.e., high tone-high tone and low tone-low tone).

#### The cued cross-modal matching trial (CCMT, Fig 1B&D)

The procedures of CCMT were identical to CUMT except (1) task cue (a cycle with dark-gray in upper half) and (2) the matching stimuli (high tone-line and low tone-curve). The participants were asked to attend the visual stimulus and ignore the auditory stimulus, and to report whether the visual S2 matches auditory S1 by pressing a button.

### Image acquisition

Imaging data were collected by a 3 T Siemens Trio MR scanner equipped with a head volume coil, with one anatomical run and four functional runs in total. The high-resolution structural image (matrix = 256×256, FOV = 240×240 mm^2^, slice thickness = 1 mm, TR = 1900 ms, TE = 3.43 ms, flip angle = 7°) for each participant was recorded using 3D MRI sequences for anatomical co-registration and normalization. Functional MRI data were obtained using a T2*-weighted echo planar imaging (EPI) sequence (FOV = 240×240 mm^2^, matrix = 64×64, in-plane resolution = 3.75×3.75 mm^2^, thickness = 4 mm, without gap, TR = 2000 ms, TE = 30 ms, flip angle = 90°).

### Image analysis

Data from 3 participants were excluded from further data analysis because of failure to accomplish the task, low behavioral performance (accuracy <70%) or serious head movements (>2 mm), respectively. Functional MRI data were analyzed by SPM8 (http://www.fil.ion.ucl.ac.uk/spm, Welcome Department of Cognitive Neurology). EPI data were first corrected for the order of slice acquisition and then realigned to the first volume within a series to correct for head motion. Next, the structural image was co-registered to the mean EPI data, segmented and generated normalized parameters to MNI space. All EPI data were then normalized to the MNI space with a resolution of 2×2×2 mm^3^ and smoothed with an 8-mm FWHM (full width half maximum) Gaussian kernel. High-pass temporal filtering with a cut-off of 128 s was also carried out to remove low-frequency drifts.

#### Whole-brain analysis

In the first level analysis, 6 task-related regressors (i.e. unimodal S1, cross-modal S1, unimodal task cue, cross-modal task cue, auditory S2 and visual S2 convolved with the canonical hemodynamic response function (HRF) were included in a general linear model (GLM), which also included 6 additional estimated parameters of head movement to rule out the effect of head motion. Statistical parameter estimates from each participant were then put into the second-level analysis based on the random-effect to allow population inference. One-sample T-test was adopted to compare the activation pattern either between different sensory modalities during the same processing phase (e.g. unimodal task cue *v.s.* cross-modal task cue) or between different processing phases in the same sensory modality (e.g. task cue *v.s.* S1). The results were reported with a voxel-wise threshold of *p*<0.001 (uncorrected) with a spatial extent threshold of *k* = 20.

#### Region-of-Interest (ROI) analysis

To further explore the activity of the task-related regions across conditions, ROI analysis was also performed via MarsBar (http://marsbar.sourceforge.net). Three ROIs was defined based on the clusters showing responses to cross-modal task cue (*v.s.* unimodal task cue) in the previous whole-brain analysis with a voxel-wise threshold of *p*<0.001 (uncorrected). The maximal MNI coordinates of these ROIs were listed as follows: left lateral prefrontal cortex (*l*-LPFC, BA 9; x/y/z = −42/10/32), left superior parietal lobule (*l*-SPL, BA 7; x/y/z = −26/−56/44), and thalamus (x/y/z = −12/−22/12). For each ROI, the percentage signal change and fitted time course in each condition of each participant was extracted, which were then put into SPSS 16.0 (Chicago, IL) for further analysis.

## Results

### Behavioral performance

**Figure 2:**
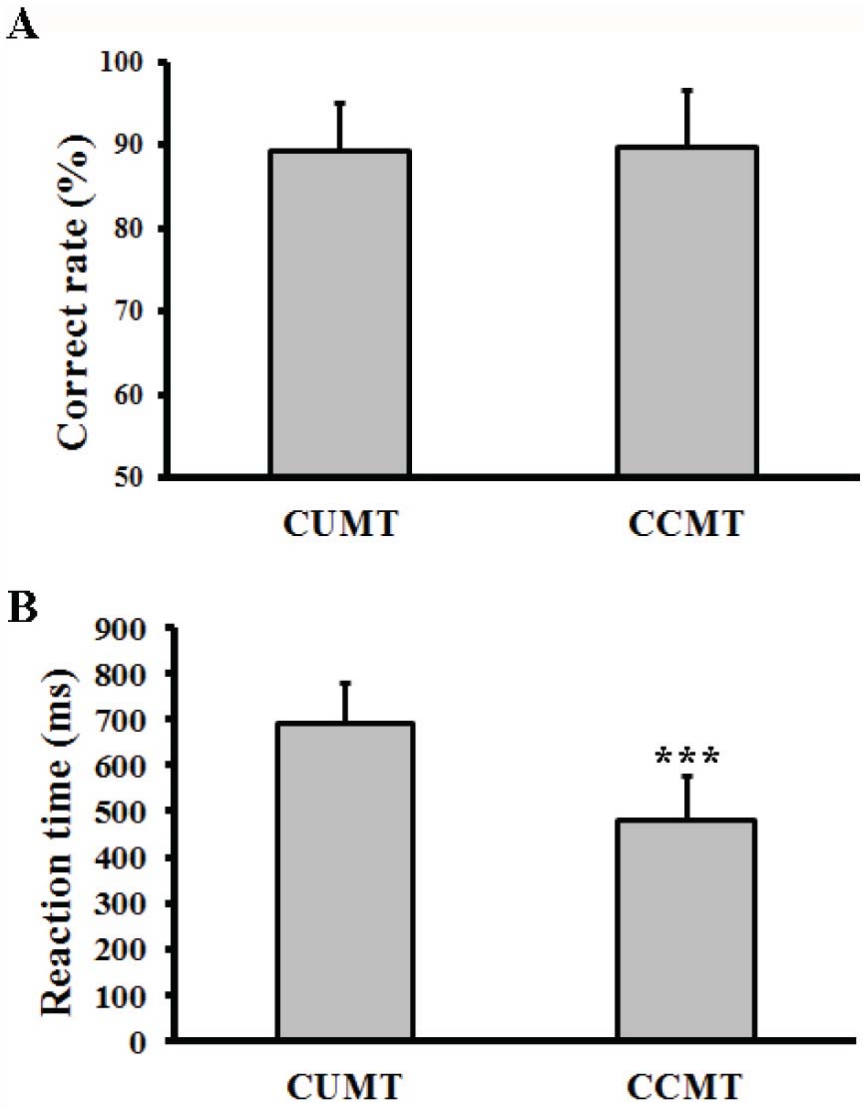
The behavioral performance of the cued matching task. *A*: Correct rate; *B*: Reaction time. CUMT indicates the cued unimodal matching trial. CCMT indicates the cued cross-modal matching trial. The error bars mean the standard deviations; ***p<0.001 (student t-test).

The averaged correct rate and reaction time in those correct trials were analyzed for both the cued unimodal matching trials (CUMT) and the cued cross-modal matching trials (CCMT). The correct rates were closed to 90% in both trials, and did not show significant difference between CUMT and CCMT trials (Fig. 2A, *p*>0.05, student t-test). The reaction times in the CCMT condition was statistically shorter than that in the CUMT condition (Fig. 2B, 480.7 ms *v.s.* 690 ms, *p*<0.001, student t-test). We did not find significant gender difference in correct rates (*t*_(15)_ = 0, *p* = 1) or reaction times (*t*_(15)_ = 1.29, *p* = 0.22). Our behavioral results revealed that cross-modal association facilitated the retrieval of memorized information.

### Neuroimaging results

#### Whole brain analysis

**Figure 3:**
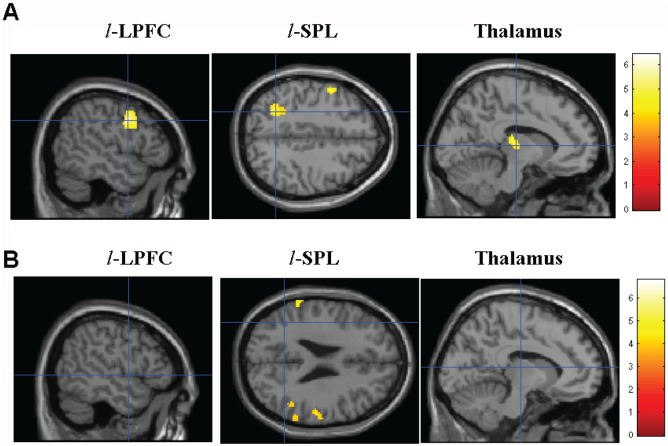
Brain regions related to initiation of cross-modal working memory retrieval (*p*<0.001, uncorrected, k = 100). *A*: the areas displaying stronger activities in CCMT trials compared to the CUMT trials; *B*: the areas displaying stronger activities in CUMT trials compared to the CCMT trials. *l*-LPFC (X = −50): left lateral prefrontal cortex; *l*-SPL (Z = 44): left superior parietal lobe; Thalamus(X = −10).

To elucidate which brain areas were involved in the initiation of cross-modal working memory retrieval, the activations in whole brain induced by the task cues were first contrasted between the CCMT and CUMT conditions (Table 1). The activations in left lateral prefrontal cortex (*l*-LPFC, BA9), left posterior parietal cortex, including superior parietal lobe (*l*-SPL, BA7) and inferior parietal lobe (*l*-IPL, BA40), and thalamus in the CCMT condition were significantly greater compared to that in the CUMT condition (Table 1 and Fig. 3A, *p*<0.001, uncorrected). However, fewer regions were found to have significantly greater task cue related activity in the CUMT condition than that in the CCMT condition (Fig. 3B).

**Table 1:** Summary of brain areas showing greater activation to task cue in CCMT than CUMT conditions (*p*<0.001, uncorrected, k = 20).

Brain areas	t	k	Hemi-sphere	MNI Coordinate			
				x	y	z	
Middle/Inferior Frontal Gyrus	6.44	419	L	−42	10	32	
Superior Parietal Lobule	5.23	266	L	−26	−58	44	
Thalamus	5.22	160	L/R	−12	−22	12	
Inferior Parietal Lobule/Sub-Gyral	4.16	79	L	−44	−36	42	
Middle Frontal Gyrus	4.21	55	L	−32	0	58	
Lingual Gyrus	4.27	46	L	−20	−90	−2	
Insula	4.54	43	R	32	−24	22	
Cingulate Gyrus	5.74	28	R	8	2	29	
Middle Frontal Gyrus	4.20	25	R	28	−6	48	

CUMT indicates the cued unimodal matching trial, CCMT indicates the cued cross-modal matching trial.

#### The ROI analysis

**Figure 4:**
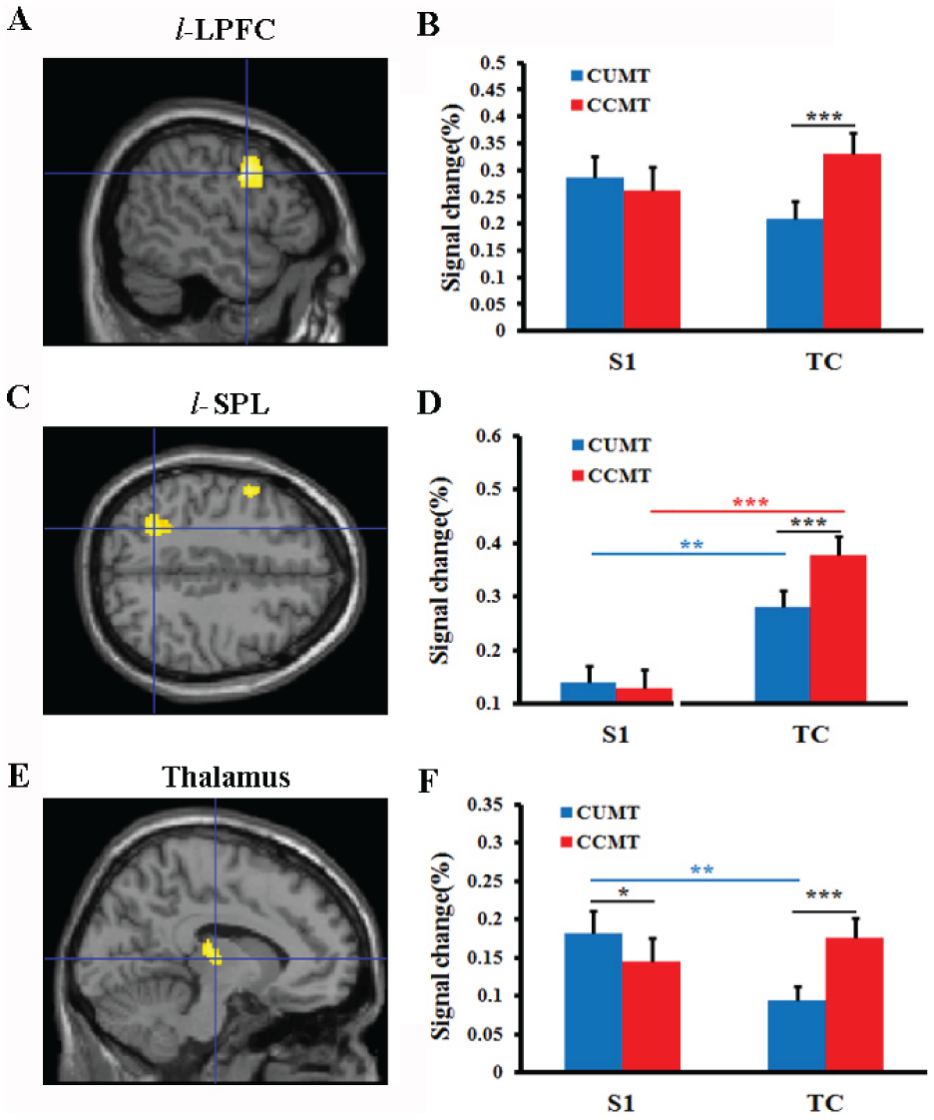
The ROI analysis of Brain activations to S1 stimulus and task. *A and B* (*l*-LPFC, X = −50): left lateral prefrontal cortex; *C and D* (*l*-SPL, Z = 44): left superior parietal lobe; *E and F* (Thalamus, X = −10); *S1*: the first stimulus (high tone or low tone) in the cued matching task; *TC*: the task cue in the cued matching task; Error bars corresponded to the standard error of mean; *p<0.05, **p<0.01, ***p<0.001 (student t-test).

Next, we functionally define ROIs in *l*-LPFC (Fig. 4A), *l*-SPL (Fig. 4C) and the left/right thalamus (Fig. 4E). The percentages of signal change produced by task cue in the CCMT trials were much higher than that in the CUMT trials in *l*-LPFC (Fig. 4B *right, student t-test, p<0.001*), *l*-SPL (Fig. 4D *right, student t-test, p<0.001*) and thalamus (Fig. 4F *right, student t-test, p<0.001*) areas. However, responses to S1 in both *l*-LPFC and *l*-SPL were not significantly different between CUMT and CCMT conditions (Fig. 4B&D). The responses in thalamus induced by task cue displayed in the opposite pattern during Delay-I (Fig. 4F).

All these data indicated that the network consisting of *l*-LPFC, *l*-SPL and thalamus might be associated with initiation of cross-modal working memory retrieval.

### Comparisons of activations between S1 stimulation and task cue

In order to examine the differential activations in these brain areas related to the maintenance of sample stimulus (S1) and initiation of working memory retrieval in the cued matching task, we compared the activations produced by task cue with the activations induced by S1 for each cued matching task. The activation of *l*-LPFC after S1 was not different from that after task cue onset in both CUMT and CCMT conditions (Fig. 4B, *student t-test, p>0.05*). However, the response to task cue in *l*-SPL was significantly higher than that to S1 in both CUMT (Fig. 4D, *student t-test, p<0.01*) and CCMT condition (Fig. 4D, *student t-test, p<0.001*). Thalamus showed reduced reactivation after task cue compared to S1 in the cued unimodal matching task (Fig. 4F, *student t-test, p<0.01*) while it did not show any difference in the cued cross-modal matching task (Fig. 4F, *student t-test, p>0.05*). Therefore, the memory maintenance and working memory retrieval might facilitate by different brain networks, especially in posterior parietal lobe and thalamus. More interestingly, the differential activation of posterior parietal lobe and thalamus in cross-modal working memory was different from those in unimodal working memory.

## Discussion

The main purpose of the present study was to investigate how the brain initiated cross-modal working memory retrieval based on task cue appeared in the middle of delay period of a cued matching task by event-related fMRI methods. We found that greater activation generated by task cues in several brain regions in cross-modal condition than that in unimodal condition, including *l*-LPFC (BA9), *l*-SPL (BA7) and bilateral thalamus. However, no difference of responses to sample stimulus (S1, auditory stimulus) was found between cross-modal and unimodal conditions. Our data indicated these brain areas might be related to the initiation of cross-modal working memory retrieval. Secondly, differential activations during Delay-II (time gap between task cue and S2) versus Delay-I (time gap between S1 and task cue) between cross-modal condition and unimodal condition were observed in both *l*-SPL and thalamus. These data indicated the differential network underlying the maintenance and memory retrieval of working memory in cross-modal working memory differed from that in unimodal working memory, which could be associated with different levels of demand for cognitive resources.

### The frontoparietal network and initiation of cross-modal working memory retrieval

The flexibility of human or animal behavior depends on the ability to choose appropriate actions according to not only the sensory information at hand but also the information retrieved from memory. In present study, participants were required to recognize the feature of sample stimulus (high or low tone) and keep it in mind during delay-I period, which was mainly related to maintenance of working memory. Then, they were asked to retrieve and expect the associated auditory stimulus in CUMT trials or visual stimulus in CCMT trials during Delay-II period, which might be mainly related to working memory retrieval. Therefore, the cognitive components during delay-I should be identical between the CCMT and CUMT trials, while cognitive components during delay-II period in CCMT trials should differ from those in CUMT trials, which was associated with initiation of cross-modal working memory retrieval controlled by task cue. Our neuroimaging results showed that frontoparietal network consisting of *l*-LPFC and *l*-SPL did display much stronger activations during Delay-II period in CCMT trials compared to in the CUMT trials while similar activations of those areas during delay-I period were obtained between these two conditions. Therefore, we proposed that the frontoparietal loops participated in the initiation of cross-modal working memory retrieval when participants performed a cued matching task. In our follow-up experiment, the S1 in the cued matching task was changed into visual stimulus (line or curve). When participants performed new cued matching task, we found that those brain areas also show greater responses to task cue in CCMT trials than in CUMT trials (data did not show here). Therefore, our data indicated that network related to initiation of cross-modal memory retrieval was independent of the modality of sample sensory information in the cued matching task.

Using single-cell recording method, several lines of evidence suggest that neuronal activity in prefrontal cortex to an identical stimulus could significantly vary as a function of which portion of that stimulus must be attended [5], the specific motor response associated with it [19] and task context [20]. Accumulating evidence has demonstrated dorsolateral prefrontal cortex is rich with rule-dependent neurons [21]. In a recent fMRI study, Chiu et al (2011) reported that a network of dorsal frontoparietal regions (left middle frontal gyrus and left inferior and superior parietal lobule) exhibited distinct patterns for race and gender discriminations of face, suggesting that these regions may represent abstract goals during high-level categorization tasks [22]. When participants performed the different stimulus-response mapping tasks according to the instruction cue (screen color) indicating which rule should be applied, Woolgar et al (2011) demonstrated that a network of frontoparietal regions (including LPFC and IPS) was associated with representation of task-relevant information [23].

The PPC is also known to play a crucial role in the integration of different modalities of stimuli [24]. When subjects were instructed to perform motion discrimination task under the simultaneous presentation of visual stimulus and tactile stimulus, the left SPL was more prominently activated under the congruent event conditions than under incongruent conditions [25], which indicating SPL involves in cross-modal integration among different sensory modalities. Using intracranial recording [26] and EEG/ERP recording [27] on humans, the SPL had been showed greater activation to multisensory stimuli than that to the sum of responses to each uni-sensory stimulus. Shomstein and Yantis (2004) demonstrated that posterior parietal and superior prefrontal cortices exhibited transient increased activity produced by the initiation of voluntary attention shifts between vision and audition [28]. These findings revealed that posterior parietal and superior prefrontal cortices played an important role in the control of cross-modal shifts of attention.

All findings above suggested that the PFC and PPC might participate in maintaining rule information in cognition task. In present study, left frontoparietal network including *l*-LPFC and *l*-SPL showed stronger response to the task cues in the cross-modal matching trials compared to the unimodal matching trials while no differential responses to S1 were found between two conditions. In addition, the activation patterns were not observed in right side of the brain. These finding suggests that left frontoparietal network might play a more important role in the initiation of cross-modal working memory retrieval compared to right lateralization of brain. This finding is consistent with previous studies, such as, Tanabe et al (2005) reported that stronger left-lateralized activation than right-side of brain when subjects completed the visuo-auditory cross-modal association learning task [14]. The finding in our study was also consistent with the idea that auditory working memory activated left lateral prefrontal cortex and left parietal cortex [15].

### Thalamus and initiation of cross-modal working memory

Converging evidence by anatomical multiple tracing methods have demonstrated there exists widely distributed thalamocortical and corticothalamic connections between different sensory and motor cortical areas and thalamic nuclei [29], which suggests the thalamus could act as a relay in multisensory processing [30]. In particular, the medial pulvinar nucleus (PuM) contains neurons projecting to the auditory cortex, the somatosensory cortex, the visual cortex, and the premotor cortex [31]. Previous studies on monkeys revealed that neurons in PuM could respond to visual stimuli [32] and auditory stimuli [33]. Therefore, the PuM is considered as the main candidate (although other thalamic nuclei may also play a role) to represent an alternative to corticocortical loops by which information can be transferred between cortical areas belonging to different sensory and sensorimotor modalities. Komura et al (2005) reported when rat performed an auditory spatial discrimination task, about 15% of neurons in the auditory thalamic nuclei displayed significantly higher discharges after simultaneous presentation of auditory and visual stimuli in the same side of animal than the sum of the unimodal responses [34]. Therefore, thalamus takes part in multisensory integration in addition to relay of sensory information through the cortico-thalamo-cortical route [35]. Using cued matching task in our present study, we found that thalamus displayed much stronger activation to task cue in CCMT trials compared to that in CUMT trials. These data indicated that thalamus might play an important role in the initiation of cross-modal memory retrieval, meanwhile we also provided the evidence for functional role of thalamus in multisensory integration. In our present study, we did not find any significant differential activation in hippocampus after appearance of task cue. Lot of evidence has shown that hippocampus is very important for acquision of memory [36], [37] and working memory [38], few study has been found so far to support hippocampus plays very important role in cross-modal working memory. Our present findings indicate again that hippocampus plays much less important role than fronto-parietal network in some higher functions (such task switching, decision making, initiation of cross-modal working memory and so on).

Previous studies have demonstrated that dyslexia [39] and autism [40] patients display a significant deficit in integration of multisensory information. The deficit in the integration of letters and speech sounds is one of causes of reading and spelling failure in dyslexia [39]. Our data provided neurofunctional evidence for potential training approach to improvements of symptoms by increased activation of brain areas related to initiation of cross-modal association.

### Differential neural responses to maintenance and memory retrieval in between cross-modal and unimodal working memory

Accumulating evidence has shown that dissociated frontoparietal networks in the brain were related to short-term maintenance and manipulation processes in spatial [18] and object working memory [17]. Moreover, schizophrenic patients performed worse than healthy controls when faced with manipulation as compared to only maintenance [41]. Mohr et al (2006) found that higher fMRI signal during the delay period of the maintenance task was observed in right superior frontal gyrus and right rostral medial frontal gyrus, while the precuneus and inferior parietal lobles displayed stronger activations during delay period of the manipulation task [42]. In our study, we proposed that main cognitive process should be maintenance of the sensory information during the Delay-I, while one of most important cognition after task cue was the retrieval of the matching stimulus based on task cue, which could be related to memory retrieval (or manipulation of working memory). Our neuroimaging result showed that much higher activations in *l*-SPL after task cue compared to Delay-I in both unimodal working memory and cross-modal working memory. Therefore, our data also suggest that *l*-SPL played a more important role in the manipulation than maintenance of working memory. More interestingly, we also found the similar dissociation in thalamus. We proposed that PPC and thalamus might be the members of the neural networks encoding different components of working memory.
